# Machine Learning-Enhanced NDIR Methane Sensing Solution for Robust Outdoor Continuous Monitoring Applications

**DOI:** 10.3390/s25247691

**Published:** 2025-12-18

**Authors:** Yang Yan, Lkhanaajav Mijiddorj, Tyler Beringer, Bilguunzaya Mijiddorj, Alex Ho, Binbin Weng

**Affiliations:** School of Electrical and Computer Engineering, University of Oklahoma, Norman, OK 73019, USA; yang5811@ou.edu (Y.Y.); lkhanaajav.mijiddorj-1@ou.edu (L.M.); tyler.beringer-1@ou.edu (T.B.); bek@ou.edu (B.M.); alex.n.ho-1@ou.edu (A.H.)

**Keywords:** CH_4_ emissions, NDIR gas sensor, field CH_4_ monitoring, machine learning, artificial neural network

## Abstract

This work presents the development of a low-cost and high-performance multi-sensory gas detection instrument named the AIMNet Sensor, with the integration of a machine learning-based data processing method. The compact and low-power instrument (8.5 × 11.5 cm, 1.4 W) houses the core sensing hardware module, Senseair K96, that integrates both a non-dispersive infrared (NDIR)-based gas sensing unit and a BME280 environmental sensing unit. To address the outdoor operation challenges caused by environmental fluctuation due to the varying temperature, humidity, and pressure, from the software aspect, multiple machine learning-based regression models were trained in this work on 13,125 calibration data points collected under controlled laboratory conditions. Among ten tested algorithms, the Multilayer Perceptron (MLP) and Elastic Net models achieved the highest accuracy, with R-squared coefficient $R^{2}>0.8$ on both indoor and outdoor scenarios, and with inter-sensor root mean square error (RMSE) within 1.5 ppm across four identical instruments. Moreover, field mobile validation was performed near a wastewater management facility using this solution, confirming a strong correlation with LI-COR reference measurements and a reliable detection of ${\mathrm{CH}}_{4}$ leaks with concentrations up to 18 ppm at the test site. Overall, this machine learning-integrated NDIR sensing solution (i.e., AIMNet) offers a practical and scalable solution towards a more robust distributed ${\mathrm{CH}}_{4}$ monitoring network for real-world field-deployable applications.

## 1. Introduction

Methane (${\mathrm{CH}}_{4}$) is the second most important anthropogenic greenhouse gas after carbon dioxide (${\mathrm{CO}}_{2}$), but contributes disproportionately to near-term climate forcing due to its strong infrared absorption bands and high radiative efficiency [1,2,3]. Although its atmospheric abundance (∼1.9 ppm) is far lower than that of ${\mathrm{CO}}_{2}$, its per-molecule warming effect is more than twenty times stronger on a 100-year timescale and over eighty times stronger on a 20-year timescale [3]. Because of its relatively short atmospheric lifetime of about 9–12 years, reducing ${\mathrm{CH}}_{4}$ emissions can yield substantial and rapid climate benefits [4]. Globally, ${\mathrm{CH}}_{4}$ emissions mainly originate from leaks and intentional releases associated with anthropogenic activities such as oil and gas operations, industrial production, and wastewater treatment [5,6]. Given its potent warming potential and short atmospheric lifetime, accurate detection and quantification of ${\mathrm{CH}}_{4}$ emissions are essential for effective climate mitigation, environmental monitoring, and regulatory compliance [7,8].

Various ${\mathrm{CH}}_{4}$ monitoring platforms have been explored and successfully deployed especially in the past decade. Among these, satellite, airborne, and ground-based observations have proven to be the most widely utilized and reliable for large-scale applications. Satellite-based instruments such as the Tropospheric Monitoring Instrument (TROPOMI) and GHGSat provide unparalleled global coverage and consistent long-term datasets for atmospheric ${\mathrm{CH}}_{4}$ retrieval [9,10]. They are effective for identifying large-scale regional emission patterns and supporting global ${\mathrm{CH}}_{4}$ inventories [11]. However, their limited spatial resolution (e.g., kilometers scale) and low revisit times (e.g., days or weeks scale) limit their ability to resolve emissions at individual facilities or transient emission events [10,12]. Airborne ${\mathrm{CH}}_{4}$ monitoring bridges the gap between satellite and ground-based systems by offering higher spatial resolution and flexible deployment. Instruments mounted on aircraft or UAVs, such as AVIRIS-NG, have detected ${\mathrm{CH}}_{4}$ plumes with meter-scale precision over industrial sites and landfills [13,14]. These platforms can operate below cloud cover and provide enhanced signal-to-noise performance relative to satellite sensors, enabling targeted surveys of specific facilities [14]. However, their operational cost, dependency on favorable weather conditions, and logistical complexity constrain them to episodic campaigns rather than continuous deployment [13].

Compared with satellite or airborne platforms, ground-based systems offer superior temporal coverage and detection sensitivity. Fixed-site analyzers, tower-based or distributed sensing networks, have been widely deployed across production fields, transmission and storage facilities, and urban areas to characterize emission variability [15,16,17,18]. Such near-field observations complement top-down remote-sensing approaches and are essential for validating regional ${\mathrm{CH}}_{4}$ inventories and evaluating mitigation effectiveness [10,18]. However, most of them require frequent calibration, maintenance, and reliable power and communication infrastructure for long-term operation [16,19]. This is particularly true when it comes to the distributed point sensors for on-site continuous monitoring purposes.

Regarding the continuous monitoring distributed sensing network, it is also worth noting that the selection of an appropriate sensing technology is essential to achieve reliable monitoring outcomes. Common ${\mathrm{CH}}_{4}$ detection principles include catalytic combustion (pellistor), metal oxide semiconductors (MOSs), thermal conductivity (TCD), and optical methods such as non-dispersive infrared (NDIR) and laser absorption spectroscopy (e.g., TDLAS, CRDS) [20,21,22,23,24]. Catalytic and MOS sensors are inexpensive and respond quickly to high ${\mathrm{CH}}_{4}$ concentrations but suffer from limited selectivity, humidity- and temperature-dependent drift, and poisoning effects that reduce their long-term stability in outdoor conditions [20]. TCD sensors quantify gas thermal properties and are robust but generally lack the sensitivity required for ambient ${\mathrm{CH}}_{4}$ monitoring and are strongly affected by environmental variations [21]. Laser-based systems, including TDLAS and CRDS, provide sub-ppm precision and excellent stability, yet their cost, size, and power consumption make them unsuitable for distributed, low-power field networks [22]. In contrast, NDIR sensors, which operate based on the Beer–Lambert absorption law near the 3.3 µm ${\mathrm{CH}}_{4}$ band, offer an optimal compromise between precision, durability, and cost for continuous outdoor operation [23,24,25]. Therefore, in comparison, the NDIR ${\mathrm{CH}}_{4}$ sensor is regarded as the preferable solution to create the ground-based distributed sensing network for outdoor infrastructure emission monitoring applications.

Nevertheless, when it comes to the outdoor ${\mathrm{CH}}_{4}$ detection, the device is still inevitably affected by environmental factors due to the fact that not only each sensing component including light source, sampling chamber and photodetectors are temperature and humidity sensitive, but also the gas absorption coefficient has a complex dependency to the environment, while these influences and challenges will be discussed in detail in the following chapter, it is pointed out that to develop an advanced data processing algorithm that can de-convolute the device’s sampled signal from such environmental dependency is critically important to mitigate environmental interferences and improve measurement accuracy. With that in mind, this work thus focuses on developing such a robust software algorithm based on machine learning models, and integrating it with a low cost NDIR ${\mathrm{CH}}_{4}$ sensing hardware, to achieve continuous and reliable outdoor measurements to support ground-based ${\mathrm{CH}}_{4}$ plume observation under diverse environmental conditions.

## 2. Methodology of the Integrated Sensing Instrument

In this section, the methodology presents the development of the machine learning-based calibration framework for a low-cost NDIR ${\mathrm{CH}}_{4}$ sensor intended for field-deployable outdoor monitoring. The methodology is divided into two parts. The first describes the hardware system, including the AIMNet gas sensing hardware design and controlled calibration setup for the machine learning training. The second part mainly focuses on the model selection and validation method for the machine learning.

### 2.1. AIMNet NDIR Gas Sensing Hardware

**Figure 1:**
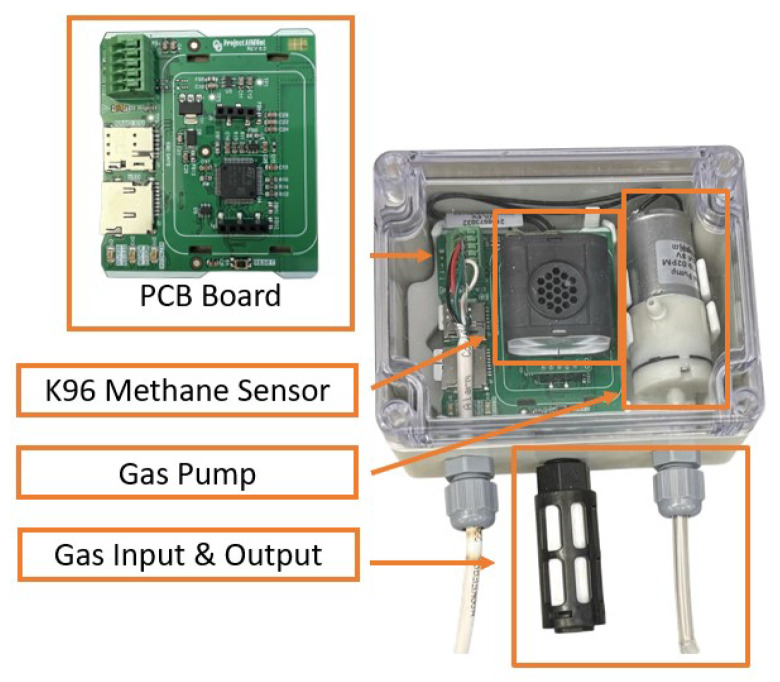
Senseair K96 NDIR ${\mathrm{CH}}_{4}$ sensor integrated in the self-designed AIMNet field device for continuous outdoor monitoring.

In terms of the hardware, the NDIR gas sensor we selected for the study is manufactured by Senseair AB (Delsbo, Sweden) called K96, within which a BME280 environmental sensor is integrated to record ambient pressure, humidity, and temperature, providing necessary environmental context for ${\mathrm{CH}}_{4}$ concentration analysis, and the physical detection limit of the K96 module is on the order of 0.5 ppm for ${\mathrm{CH}}_{4}$. This K96 functions as a core sensing module within our fully operational gas monitoring instrument. As illustrated in Figure 1, our complete sensor device was designed and assembled around this K96 module together with a custom embedded PCB control board. The embedded controller, based on an STM32U083RCT6 microcontroller mounted directly on the PCB, eliminates unnecessary I/O ports and unused circuitry that commercial microcontroller development boards usually carry, thereby maintaining the overall system integrity while reducing both cost and power consumption. Specifically, the instrument measures 8.5 × 11.5 cm and houses all components within an environmentally sealed enclosure for extended field deployment. The total power consumption is approximately 1.4 W, which enables long-term autonomous operation when powered by a solar panel system in remote outdoor environments. In addition, an air-inlet filter was installed at the sampling port to prevent dust and insect ingress, ensuring reliable performance and sensor longevity in outdoor conditions. To ensure a stable and sufficient gas intake, we integrated an air pump (ZR370-02PM), which can provide up to 2.5 L $\min^{-1}$ flow rate to capture sufficient sample gas when needed. The pump flow rate is controlled via pulse-width modulation (PWM), allowing us to adjust the sampling flow for different operating modes, such as long-term and short-term monitoring, power-saving mode, and driving mode. For data communication and remote control, the device incorporates a SIM7070G cellular modem and an on-board LTE antenna (model A1004795), enabling near real-time transmission of ${\mathrm{CH}}_{4}$ measurements and device status to a central server when needed. The sensor is sampled internally at 1 Hz, while the data-upload interval is configurable and can be set from 1 Hz down to much slower reporting rates (e.g., ten minutes or hours) depending on power and bandwidth constraints. This architecture allows near real-time signal processing (within 1 ms per data), data management, and GIS-based visualization to be performed in the cloud rather than being limited by the on-board hardware. A microSD card slot is also integrated inside the device to provide local data backup in case of temporary network or data-transmission failures, and the entire electronics are implemented using surface-mount technology (SMT) to improve robustness and manufacturability [26,27,28].

**Figure 2:**
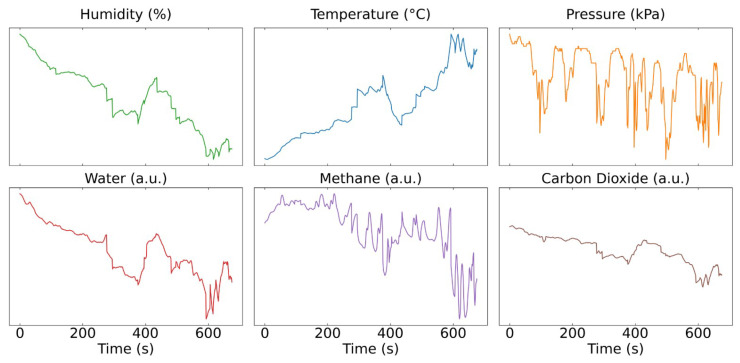
Field test data from the integrated ${\mathrm{CH}}_{4}$ sensing system. The **upper panel** shows temperature, humidity, and pressure recorded by the BME280 sensor, and the **lower panel** displays the raw infrared absorption signals of H_2_O, ${\mathrm{CH}}_{4}$, and CO_2_ measured by the K96 sensor.

The integrated system acquires data from the K96 CH_4_ sensor at a frequency of 1 Hz. Figure 2 presents one representative dataset collected during a field test. The left panel displays the BME280 measurements of temperature, humidity, and pressure, while the right panel shows the raw infrared absorption signals corresponding to H_2_O, ${\mathrm{CH}}_{4}$, and ${\mathrm{CO}}_{2}$ channels. The observed signal variation is considerably larger than what would be expected for accurate gas concentration readings, indicating that the raw sensor output could be influenced by environmental factors. From Figure 2, it can be seen that the H_2_O channel strongly follows the trend of humidity and temperature changes, and the CH_4_ channel partially follows the variations of the H_2_O signal and ambient pressure. It is obvious that even though the device is placed inside a sealed and water proof enclosure, its optical measurements are still affected by surrounding environmental fluctuations.

To understand this environmental impact, We can start with the operating principle of the NDIR CH_4_ sensor—the Beer–Lambert law, which describes the exponential attenuation of infrared radiation as it passes through an absorbing medium. Based on the Beer–Lambert Law, the relationship between gas concentration and transmitted light intensity can be expressed as

(1) $$I=I_{0}\exp\left(-\alpha \;c\;L\right)$$

In practice, the absorption coefficient α of ${\mathrm{CH}}_{4}$ is not constant, as it varies with gas temperature, pressure, and the presence of interfering absorbers such as water vapor molecules (H_2_O) [22,29,30,31]. Temperature changes modify the molecular population distribution and consequently alter the absorption line strength, while humidity introduces overlapping absorption bands near the CH_4_ fundamental at 3.3 µm, leading to cross-sensitivity and baseline drift. As a result, uncorrected variations in environmental temperature and humidity can distort the measured transmittance $I/I_{0}$ and produce biased CH_4_ readings. Furthermore, the performance of each sensing components within the NDIR module including light source, sampling chamber and photodetectors are of temperature or humidity dependency. With all these being considered, the fluctuations of the CH_4_ signal observed in the field measurements is therefore of a strong tie back to the environmental temperature and humidity fluctuations. As a result, implementing effective temperature and humidity compensation algorithm is essential to minimize such environmental influences and ensure accurate CH_4_ quantification in outdoor conditions.

It is important to note that, as shown in Figure 2, the CH_4_ signal exhibits a very complicated non-linear relationship with temperature and humidity for this sensor. Moreover, the magnitude of the environmental noise is significantly larger than the variation in the CH_4_ signal itself, making it difficult to isolate and remove such interference using conventional signal-processing techniques. Several recent studies have demonstrated the successful application of machine learning methods in gas sensing tasks, achieving improved prediction accuracy under varying environmental conditions [32,33]. However, limited research has specifically focused on applying such approaches to NDIR-based CH_4_ sensing. Motivated by these findings, in this research, multiple machine learning models commonly used in gas sensing applications were implemented and evaluated to determine whether they could effectively compensate for environmental interferences and improve CH_4_ concentration estimation accuracy.

### 2.2. Data Calibration

To correctly and comprehensively analyze the sensor data, it is essential to first obtain a sufficiently large dataset that captures a wide range of outdoor environmental conditions. Several similar experimental setups for NDIR-type gas sensors have been reported in previous studies [32,33]. The most important requirement is to establish a controlled experimental environment in which the gas concentration, humidity, and temperature can be systematically varied.

**Figure 3:**
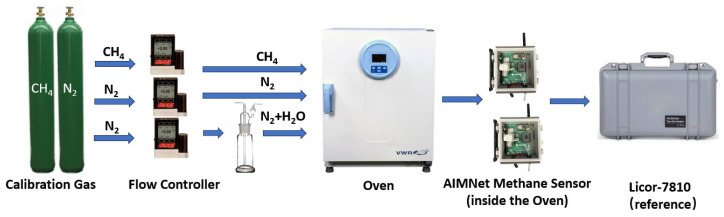
Custom-built environmental chamber controlling ${\mathrm{CH}}_{4}$ concentration, relative humidity, and temperature for NDIR sensor calibration experiments.

Figure 3 illustrates the laboratory setup used for data collection to evaluate the performance of the CH_4_ sensor under different environmental conditions. To get the absolute value of the CH_4_ reading, we mixed the CH_4_ and N_2_ calibration gas for data collection. As shown in the diagram, two mass flow controllers (MFCs) from Alicat Scientific (Tucson, Arizona, USA) are employed to regulate the flow rates of CH_4_ and N_2_ calibration gases, thereby controlling the CH_4_ concentration in the test mixture. The calibration gases provide a dry and high-purity baseline. One of the gas lines is passed through a water bubbler to increase the humidity level, while the ratio between the humidified and dry gas streams determines the relative humidity of the gas mixture entering the sensor chamber. The AIMNet ${\mathrm{CH}}_{4}$ sensor is then connected downstream and placed inside a temperature-controlled oven to precisely adjust the testing temperature. Furthermore, as illustrated in the figure, the gas outlet of the AIMNet sensing unit was directly connected to a high-precision LI-COR 7810 analyzer (LI-COR Biosciences, Lincoln, NE, USA), which served as the reference instrument for accurate ${\mathrm{CH}}_{4}$ concentration measurements, with a nominal precision of approximately 0.25 ppb (1σ) at ambient ${\mathrm{CH}}_{4}$ levels.

**Figure 4:**
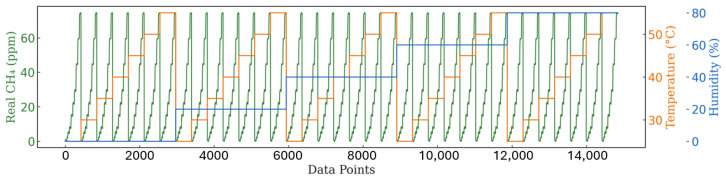
Overview of calibration dataset covering variations in humidity, temperature, and ${\mathrm{CH}}_{4}$ concentration.

To maximize the effectiveness of the data analysis, a comprehensive dataset was constructed to cover a wide range of environmental conditions. As shown in Figure 4, the dataset includes measurements collected under five distinct humidity levels, with relative humidity ranging from 0% to 80%. For each humidity condition, tests were conducted at five temperature settings between 25 °C and 55 °C, with an increment of 5 °C. Within each temperature and humidity combination, the ${\mathrm{CH}}_{4}$ concentration was gradually varied from 0 to 15 ppm in 3 ppm increments, allowing several minutes at each step to ensure signal stabilization. Subsequently, the concentration was increased from 15 to 65 ppm in 10 ppm steps. In total, over 15,000 data points were recorded during the calibration process.

### 2.3. Data Preprocessing and Split

Before model training, several preprocessing steps were applied to ensure data quality and consistency. First, all records containing invalid or corrupted sensor outputs were removed, including communication errors, negative readings, and physically impossible values. Data collected during the sensor warm-up period were also discarded, as the NDIR module exhibits unstable absorption signals immediately after powering on. In addition, continuously repeated data points were filtered out to prevent oversampling of identical values. These preprocessing steps discard only a small fraction of the data. And based on inspection of the ${\mathrm{CH}}_{4}$ and environmental-variable histograms before and after filtering, these steps do not materially alter the underlying data distributions.

After filtering out invalid signals and duplicate entries, a total of 13,125 valid data points were retained for model development. To ensure fairness and avoid temporal bias, the complete dataset was randomly shuffled before splitting, and all normalization parameters were computed strictly from the training subset to prevent information leakage. All models were trained with fixed random seeds for reproducibility, and their performance was evaluated using multiple datasets described below.

The shuffled dataset was then randomly divided into two subsets: 80% for training (*Data-train*) and 20% for validation (*Data-val*). Because the calibration dataset was collected using a pure $N_{2}$ carrier gas, additional tests were conducted to assess model robustness under more realistic conditions. Two separate test datasets were created for this purpose. The first test set (*Data-test1*) corresponds to indoor measurements using a pump to deliver ${\mathrm{CH}}_{4}$ calibration gas, while the second test set (*Data-test2*) was collected outdoors under stable environmental conditions using portable ${\mathrm{CH}}_{4}$ calibration gas. In all experiments, the reference analyzer provided the ground-truth ${\mathrm{CH}}_{4}$ concentration values.

### 2.4. Performance Evaluation

According to the U.S. Environmental Protection Agency’s (EPA) recommended performance evaluation framework for low-cost air sensors [34], the performance of the developed ${\mathrm{CH}}_{4}$ sensor was assessed using two widely adopted quantitative metrics: the root-mean-square error (RMSE) and the coefficient of determination ($R^{2}$). These metrics are defined as

(2) $$\mathrm{RMSE}=\sqrt{\frac{1}{n}\sum_{i=1}^{n}{\left({\hat{y}}_{i}-y_{i}\right)}^{2}}$$

(3) $$R^{2}=1-\frac{\sum_{i=1}^{n}{\left(y_{i}-{\hat{y}}_{i}\right)}^{2}}{\sum_{i=1}^{n}{\left(y_{i}-\bar{y}\right)}^{2}}$$

where $y_{i}$ represents the reference measurements, ${\hat{y}}_{i}$ denotes the sensor-estimated concentrations, and $\bar{y}$ is the mean of the reference data. To ensure external validity beyond controlled laboratory conditions, collocated field measurements were conducted in two distinct outdoor environments. Multiple identical sensing units were deployed in parallel to quantify unit-to-unit variability, while a high-precision reference analyzer provided traceable ground-truth ${\mathrm{CH}}_{4}$ concentrations.

### 2.5. Machine Learning Algorithm

To comprehensively evaluate the performance of various machine learning models on the regression task for this ${\mathrm{CH}}_{4}$ sensor, we tested more than ten widely used algorithms that have shown potential in gas-sensing and environmental-prediction applications. The models exhibiting the best overall performance, as well as those employing specialized learning mechanisms, are summarized below.

#### 2.5.1. Multiple Linear Regression

Multiple Linear Regression (MLR) is a fundamental statistical approach used to describe the relationship between one dependent variable and several independent variables. It assumes that the target gas concentration can be represented as a linear combination of multiple predictors, such as sensor signals and environmental factors (temperature, humidity, and pressure). The model can be written as

(4) $$y=\beta_{0}+\beta_{1}x_{1}+\beta_{2}x_{2}+\cdots +\beta_{n}x_{n}+\epsilon ,$$

where *y* is the predicted concentration, $x_{i}$ are the input features, $\beta_{i}$ are the regression coefficients, and ϵ is the residual error. MLR offers a transparent and computationally efficient framework for gas-sensor calibration, allowing environmental parameters to be directly integrated for compensation. Previous studies have demonstrated that applying MLR to low-cost or NDIR-based gas sensors can effectively improve accuracy and stability in variable outdoor environments [35,36,37].

#### 2.5.2. Elastic Net Regression

Elastic Net Regression (ENR) is a regularized linear modeling approach that integrates the strengths of both Ridge ($L_{2}$) and Lasso ($L_{1}$) regression [38]. It overcomes the limitations of traditional linear models in handling correlated predictors by combining variable selection and coefficient shrinkage within a unified framework. The objective function of the Elastic Net can be formulated as

(5) $$L\left(\beta \right)={∥y-X\beta ∥}_{2}^{2}+\lambda_{1}{∥\beta ∥}_{1}+\lambda_{2}{∥\beta ∥}_{2}^{2},$$

where *y* represents the vector of observed values, *X* is the predictor matrix, β is the coefficient vector, and $\lambda_{1}$ and $\lambda_{2}$ are regularization parameters controlling the $L_{1}$ and $L_{2}$ penalties, respectively. This hybrid penalty encourages sparse feature selection while maintaining stability in the presence of multicollinearity. Due to its balance between interpretability and predictive power, Elastic Net has become one of the most commonly used linear regularization techniques in statistical learning and data-driven modeling.

#### 2.5.3. Support Vector Regression

Support Vector Regression (SVR) is an extension of the Support Vector Machine (SVM) framework for solving regression problems [39,40]. It aims to find a regression function that fits the data within a predefined tolerance while maintaining maximum flatness. The standard linear SVR model is expressed as

(6) f(x)=〈w,x〉+b,

where *w* is the weight vector and *b* is the bias term. The optimization seeks to minimize ${∥w∥}^{2}$ under the constraint

(7) $$|y_{i}-f\left(x_{i}\right)|\leq \epsilon ,$$

where ϵ defines the maximum allowable deviation from the true values. By using kernel functions such as the radial basis function (RBF), SVR can efficiently capture nonlinear relationships between input variables and the target gas concentration while preserving high generalization ability. SVR is particularly advantageous in environmental sensing tasks due to its robustness to noise and its ability to handle complex, multidimensional feature spaces.

#### 2.5.4. CatBoost Regression

CatBoost Regression (CBR) is a gradient boosting algorithm that builds an ensemble of decision trees while efficiently handling categorical features and reducing prediction bias [41,42]. It extends the principles of gradient boosting by introducing an ordered boosting mechanism that prevents target leakage and overfitting when training with categorical data. The model iteratively constructs weak learners to minimize a differentiable loss function, typically expressed as

(8) $$L=\sum_{i=1}^{n}\ell \left(y_{i},f_{m-1}\left(x_{i}\right)+\gamma_{m}h_{m}\left(x_{i}\right)\right),$$

where *ℓ* is the loss function, $f_{m-1}$ is the current ensemble model, $h_{m}$ is the newly added tree, and $\gamma_{m}$ is the learning rate controlling update strength. CatBoost employs symmetric (oblivious) trees and an advanced encoding scheme for categorical features, enabling it to deliver high accuracy even with limited data preprocessing. It provides strong generalization performance, excellent handling of non-linear dependencies, and fast convergence, making it particularly suitable for environmental prediction and sensor calibration tasks where data are heterogeneous and partially categorical in nature.

#### 2.5.5. Random Forest Regression

Random Forest Regression (RFR) is an ensemble learning algorithm that constructs a large number of decision trees and combines their predictions through averaging to improve accuracy and reduce overfitting [43]. Each tree in the forest is trained on a bootstrap sample of the original dataset, and at each split, a random subset of features is selected to ensure model diversity. The regression output of the ensemble is given by

(9) $$\hat{y}=\frac{1}{N}\sum_{i=1}^{N}f_{i}\left(x\right),$$

where *N* is the number of trees and $f_{i}\left(x\right)$ represents the prediction from the *i*th decision tree. By aggregating multiple weak learners, RFR achieves high predictive stability, robustness to noise, and the ability to model nonlinear relationships between variables. Its interpretability through feature-importance analysis and resilience to multicollinearity make it particularly suitable for environmental data modeling and gas-sensor calibration, where complex interactions often exist among temperature, humidity, and sensor responses.

#### 2.5.6. Multilayer Perceptron Regression

Multilayer Perceptron (MLP) regression is a feedforward artificial neural network model capable of approximating complex nonlinear mappings between input and output variables [44,45]. An MLP consists of an input layer, one or more hidden layers, and an output layer, where each neuron applies a nonlinear activation function to a weighted sum of its inputs. The model can be expressed as

(10) $$\hat{y}=f\left(x\right)=\sigma \left(W_{2}\;\phi \left(W_{1}x+b_{1}\right)+b_{2}\right),$$

where $W_{1},W_{2}$ and $b_{1},b_{2}$ are the weight matrices and bias vectors, ϕ(·) is the activation function (e.g., ReLU or tanh), and σ(·) denotes the output mapping function. During training, the model parameters were optimized using the backpropagation algorithm to minimize a loss function such as mean squared error (MSE). Because of its ability to capture nonlinear dependencies and interactions among multiple input variables, MLP regression provides superior flexibility for modeling sensor data and compensating for environmental variations in complex real-world scenarios.

## 3. Results and Discussion

### 3.1. Training and Validation

To obtain a comprehensive and reliable assessment, all models were trained using the cleaned and standardized dataset described previously. Hyperparameters for the linear models (MLR and Elastic Net), Support Vector Regression, Random Forest, and CatBoost were tuned through grid search or built-in cross-validation routines, while the Multilayer Perceptron (MLP) network was trained using the Adam optimizer with tuned learning rate, weight decay, and dropout rate. We monitored the prediction performance on both *Data-Val* and *Data-Test1 during training*. An early-stopping strategy was employed for the neural network to prevent overfitting and training drift: once the monitored metric on the validation stream began to deteriorate (i.e., a sustained decrease in $R^{2}$ or an increase in the validation loss), training was halted and the best checkpoint was retained. This joint monitoring of *Data-Val* and *Data-Test1* ensures that the selected models do not overfit to a single split while still capturing stable structure in the data. After systematic hyperparameter exploration and feature-combination screening, we applied a selection criterion requiring each candidate to achieve $R^{2}>0.8$ on *both Data-Val* and *Data-Test1*. Models satisfying this threshold were then ranked by stability and accuracy, yielding six top-performing configurations summarized in Table 1.

To further verify robustness under varying environmental conditions (e.g., outdoor variability and potential covariate shift), we subsequently evaluated the shortlisted models on *Data-Test2* as an out-of-distribution (OOD) test set. This additional probe is designed to confirm whether gains observed on *Data-Val* and *Data-Test1* translate to stronger generalization beyond the training regime.

**Figure 5:**
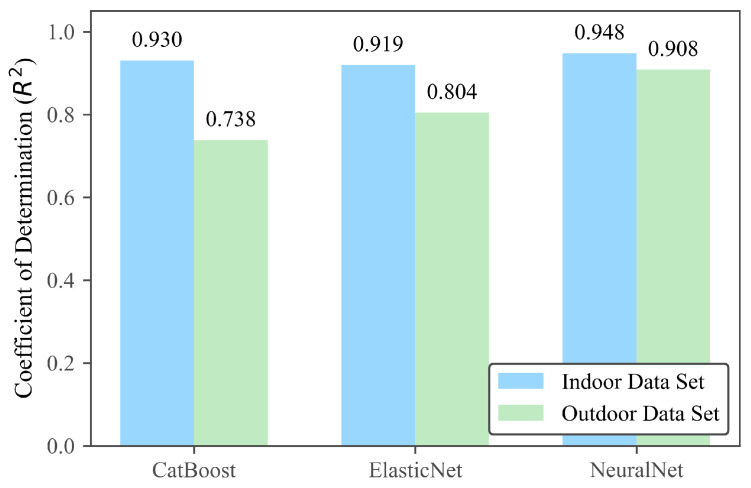
Training results comparison among the top three models: CatBoost (**left**), Elastic Net Regression (**middle**), and Multilayer Perceptron (MLP) Neural Network (**right**).

Even though all models performed remarkably well on *${\mathrm{Data-Test}}_{1}$*, their accuracy consistently decreased when evaluated on *${\mathrm{Data-Test}}_{2}$*, with several models even dropping below an $R^{2}$ value of 0.8. This decline indicates that the outdoor dataset contains data points outside the training range, exposing limitations in each model’s ability to extrapolate under unseen environmental conditions. Among the tested algorithms, the Random Forest (RFR) and Multiple Linear Regression (MLR) models exhibited the lowest performance, with $R^{2}$ values falling below 0.7 on *${\mathrm{Data-Test}}_{2}$*, confirming their restricted capability to capture nonlinear and cross-dependent environmental effects. Support Vector Regression (SVR) and CatBoost Regression (CBR) demonstrated noticeable improvements compared to the baseline models; however, CatBoost still experienced a moderate degradation under outdoor conditions, while SVR remained less stable overall, with $R^{2}$ values only slightly above 0.7 on *${\mathrm{Data-Test}}_{2}$*. As illustrated in Figure 5, CatBoost, Elastic Net, and MLP were selected as the top three models based on their combined performance on *${\mathrm{Data-Test}}_{1}$* and *${\mathrm{Data-Test}}_{2}$*, and represent the overall best performers, displaying the highest predictive accuracy across multiple environments.

The CatBoost model was trained using a refined configuration with parameters: depth = 8, learning rate = 0.055, iterations = 4000, subsample = 0.8, L2 leaf regularization = 3.0, border_count = 254, and grow_policy = Lossguide. This gradient-boosting ensemble effectively captured nonlinear dependencies among the sensor features. The resulting model achieved an $R^{2}$ of approximately 0.9304 on *${\mathrm{Data-Test}}_{1}$* and 0.7384 on *${\mathrm{Data-Test}}_{2}$*.

The Elastic Net regression model combined a StandardScaler preprocessor with ElasticNetCV regularization, using parameters: L1_ratio = [0.2, 0.4, 0.6, 0.8, 0.95], cross-validation = 5 folds, and a maximum of 5000 iterations. This hybrid regularization approach efficiently balanced bias and variance while maintaining interpretability. It achieved an $R^{2}$ of ≈0.9194 on *${\mathrm{Data-Test}}_{1}$* and ≈0.8042 on *${\mathrm{Data-Test}}_{2}$*.

The feedforward Multilayer Perceptron (MLP) network consisted of two fully connected hidden layers with 256 and 128 neurons, respectively, each followed by ReLU activation and a dropout layer (p=0.12). The model was trained using the Adam optimizer with a learning rate of $8\times 10^{-4}$, weight decay of $3\times 10^{-4}$, batch size = 256, and an early-stopping patience of 35 over a maximum of 550 epochs. The MLP achieved excellent predictive accuracy, with $R^{2}\approx 0.9480$ on *${\mathrm{Data-Test}}_{1}$* and $R^{2}\approx 0.9076$ on *${\mathrm{Data-Test}}_{2}$*, demonstrating strong generalization under variable environmental conditions.

Based on the comparative results, the top two models Elastic Net and MLP were selected for further validation, as they exhibited strong and consistent performance across both datasets. In contrast, the CatBoost model achieved an $R^{2}$ below 0.8 on the outdoor dataset, indicating insufficient generalization under real-world conditions; therefore, it was excluded from subsequent analysis. The following section focuses on validating and evaluating the performance of the selected models under various environmental scenarios.

According to the U.S. EPA’s performance recommendations for air sensors, it is essential to ensure consistent behavior across all sensing units. To verify the model’s general applicability, we validated its performance using four AIMNet ${\mathrm{CH}}_{4}$ sensors fabricated under identical configurations. Each sensor was tested following the same experimental procedure illustrated in the block diagram and was connected to the LI-COR reference analyzer for accuracy assessment. The resulting RMSE values were used to quantify the prediction error of each sensor.

**Figure 6:**
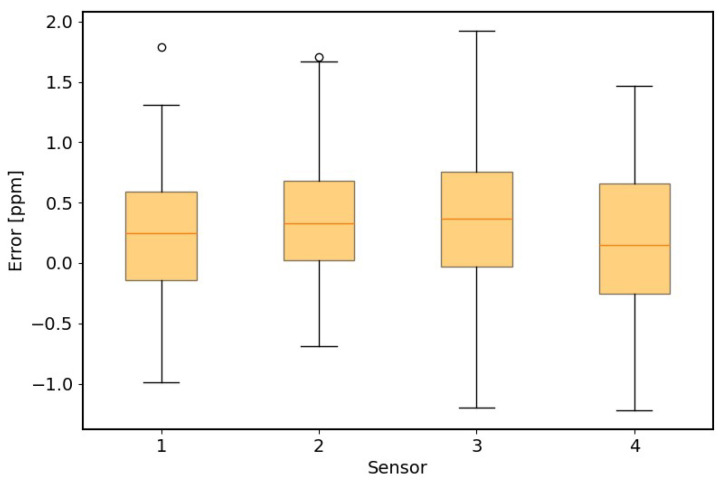
Boxplot of ${\mathrm{CH}}_{4}$ prediction residuals from four identical AIMNet sensors using the MLP model. Each box shows the distribution of prediction errors relative to the LI-COR reference measurements.

As shown in Figure 6, the MLP model outputs indicate that the four sensors exhibit slightly different absolute readings but maintain highly consistent response trends. The overall prediction errors for all units remain within 1 ppm, confirming excellent model stability and sensor-to-sensor reproducibility. The Elastic Net regression model showed slightly higher deviations, with overall RMSE values remaining within 1.5 ppm, but still demonstrated consistent performance across all sensors; therefore, only the MLP results are presented for brevity.

These results confirm that the proposed calibration framework can be reliably applied to multiple AIMNet units, ensuring stable and transferable performance in field deployments.

### 3.2. Outdoor Validation Result

**Figure 7:**
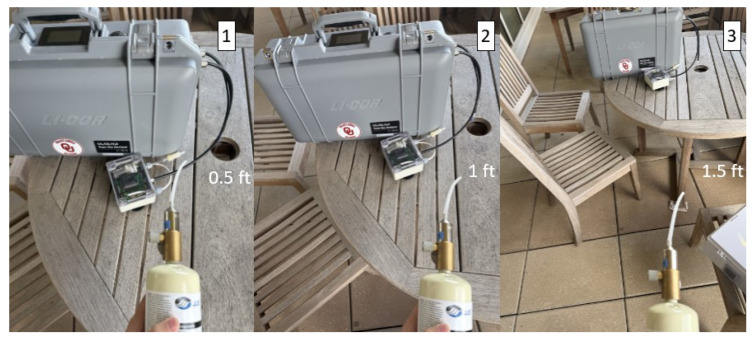
Balcony Validation Test of the AIMNet Sensor Against the LI-COR 7810 at Three Separation Distances (0.5 ft, 1 ft, and 1.5 ft).

To validate the performance of the developed ${\mathrm{CH}}_{4}$ sensor, a series of outdoor experiments were conducted under real environmental conditions. The device was deployed on the balcony outside our laboratory, as shown in Figure 7. To obtain accurate reference measurements, the sensor output was continuously connected to the input of a LI-COR reference analyzer.

To evaluate the sensor’s capability to detect ${\mathrm{CH}}_{4}$ emissions at different distances, portable calibration gas cylinders were used to release ${\mathrm{CH}}_{4}$ at multiple positions relative to the device. The test was performed at distances of 0.5 ft, 1 ft, and 1.5 ft, with calibration gas concentrations of 10 ppm, 50 ppm, and 100 ppm. Several environmental scenarios were examined, as summarized in Table 2. The experiments were carried out during spring in Norman, Oklahoma, where daytime temperatures reached approximately 28 °C, while nighttime temperatures dropped to 0–5 °C. Additional tests were conducted on rainy days (high humidity) and during a thunderstorm event characterized by high wind speed and rapidly fluctuating pressure.

From the results summarized in Table 2, the sensor demonstrated stable and accurate performance under clear weather conditions, regardless of daytime or nighttime testing. The nighttime RMSE increased slightly, likely due to temperatures below 20 °C, outside the model’s original training range, which caused minor variance in the predictions. Under rainy conditions, the Elastic Net model showed greater sensitivity to humidity changes, with the RMSE increasing to 2.73. During the thunderstorm test, the Elastic Net RMSE further increased to 3.91, suggesting that rapid variations in humidity and atmospheric pressure may have affected the sensor readings.

**Figure 8:**
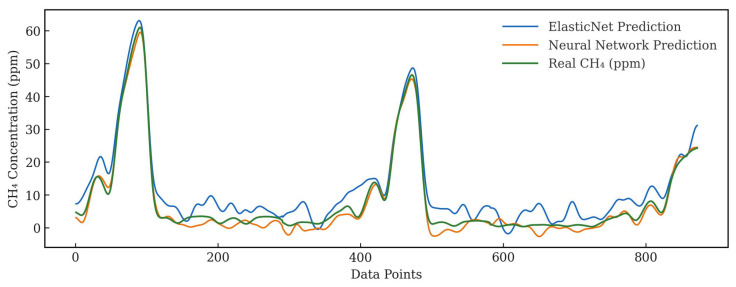
Comparison of ${\mathrm{CH}}_{4}$ concentration readings from the developed sensor and the LI-COR reference instrument under thunderstorm conditions. The plot shows predicted ${\mathrm{CH}}_{4}$ concentrations from the Elastic Net and MLP models alongside reference measurements, illustrating the sensor response to varying emission levels and environmental fluctuations.

From Figure 8, it can be observed that although the RMSE values are relatively high, both prediction models successfully follow the trend of the reference LI-COR sensor readings. The models are able to capture large ${\mathrm{CH}}_{4}$ emission events across all tested distances. The observed decrease in concentration with increasing distance is likely due to ${\mathrm{CH}}_{4}$ dilution in ambient air, as the released amount was insufficient to maintain a stable plume. The strong correlation between the predicted and reference readings indicates that the models maintain good overall performance under these dynamic outdoor conditions.

The Elastic Net model exhibited noticeable fluctuations that did not correspond to real ${\mathrm{CH}}_{4}$ variations, suggesting potential sensitivity to changes in atmospheric pressure or other environmental factors. In addition, both models showed a small offset relative to the LI-COR reference, implying that humidity effects were not fully compensated. In contrast, the Neural Network (MLP) model demonstrated smoother predictions with fewer spurious fluctuations and better agreement with the LI-COR reference readings.

### 3.3. Driving Test Result

Since the ultimate goal of this work is to develop an application-ready device for real-world ${\mathrm{CH}}_{4}$ monitoring in oil and gas fields, the system must be capable of detecting emissions from considerable distances relative to actual leakage sources. The City of Norman Water Reclamation Facility, located near the University of Oklahoma campus, continuously emits small amounts of ${\mathrm{CH}}_{4}$ as a byproduct of its wastewater treatment processes. Therefore, this site was selected as a suitable field-testing location for outdoor validation.

**Figure 9:**
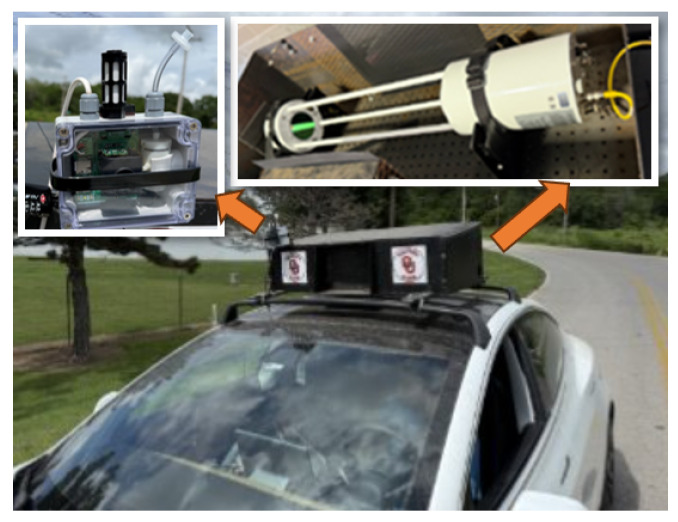
Driving Test Setup: The **left** panel shows the AIMNet sensor mounted on the mobile test platform, while the **right** panel displays the LI-COR 7700 reference instrument used for performance comparison.

To perform the field measurement, the AIMNet sensor was mounted on the roof of a vehicle, as shown in Figure 9, to capture ${\mathrm{CH}}_{4}$ emissions while driving near the facility. Similar to the laboratory and balcony validation tests, a high-precision reference instrument was used to verify measurement accuracy. The LI-COR 7700 open-path ${\mathrm{CH}}_{4}$ analyzer, with ppb-level sensitivity (nominal precision of about 5 ppb at ambient ${\mathrm{CH}}_{4}$ levels), served as the reference sensor. To ensure both instruments sampled the same air mass, the inlets of the LI-COR and AIMNet systems were positioned in close proximity. Furthermore, to preserve high-frequency measurements without using transmission intervals, we configured the system to upload data once every hour. The field measurements were performed in the early morning, with the vehicle traveling at an average speed of 15 mph around the facility area and approximately 50 mph on the highway located at the upper boundary of the site. For consistency, the pump flow rate of the AIMNet sensor is set to 250 sccm, identical to the flow rate used for the LI-7810 in our previous tests [46].

**Figure 10:**
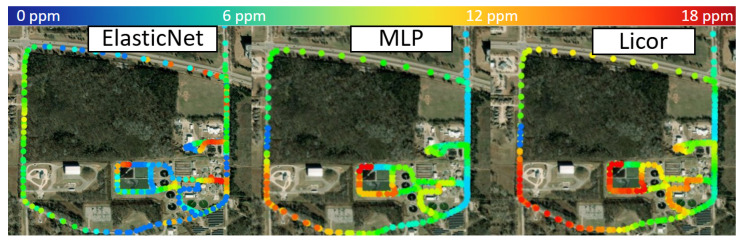
Comparison of ${\mathrm{CH}}_{4}$ concentration distributions from GIS plots for the Elastic Net model, the MLP Neural Network, and the LI-COR reference instrument during the mobile driving test.

As shown in Figure 10, which presents the GIS plot of the entire driving test, the wastewater treatment facility is located in the southeast corner of the map. During the test, the prevailing wind direction was toward the southwest, meaning that elevated ${\mathrm{CH}}_{4}$ concentrations were expected in that region. Consistent with this expectation, the LI-COR reference readings indicated higher ${\mathrm{CH}}_{4}$ levels—reaching approximately 18 ppm around the southwest corner, while concentrations near the facility ranged from 3 to 5 ppm and remained around 2–3 ppm elsewhere, representing the background level of ambient air.

The MLP model successfully captured all major ${\mathrm{CH}}_{4}$ peaks that coincided with the southwest corner of each driving path, closely matching the reference data. However, it missed several minor emission peaks in the 5–10 ppm range, which are represented by orange and yellow regions on the map. The Elastic Net regression model exhibited weaker performance, detecting significant peaks only within the facility boundaries while failing to capture elevated readings along the southwest road. Based on the results of the previous balcony test, this degradation between the AIMNet and open-path measurements can likely be attributed to a combination of pressure fluctuations associated with vehicle speed variations and structural differences between the two systems. The open-path analyzer directly samples the passing ${\mathrm{CH}}_{4}$ plume, whereas the AIMNet sensor draws air through an enclosed inlet with a relatively low pump flow rate. Under certain wind directions, the ${\mathrm{CH}}_{4}$ plume may not be efficiently captured by the AIMNet inlet, so that the open-path sensor records strong enhancements while the AIMNet system measures only background air, leading to a bias between the two instruments. A point-to-point comparison with the reference measurements shows that the RMSE of the Elastic Net model increased to 4.73 ppm, whereas the MLP model exhibited a smaller increase to 2.98 ppm. Overall, the MLP model demonstrated robust performance in detecting large emission events, though both models displayed limitations when operating under high-speed driving conditions, such as on highways.

## 4. Conclusions

This study presented the design, calibration, and validation of a compact, low-power NDIR-based ${\mathrm{CH}}_{4}$ sensing system (AIMNet) for continuous ground-based monitoring in real-world environments such as oil and gas fields and wastewater facilities. The system integrates a Senseair K96 CH_4_ sensing module within an environmental-proof, energy-efficient enclosure. Through machine learning-based calibration, particularly using Elastic Net and Multilayer Perceptron (MLP) models, the sensor achieved consistent performance with $R^{2}>0.8$ across both indoor and outdoor datasets and inter-sensor variations within 1–1.5 ppm. Field and mobile tests verified reliable detection of ${\mathrm{CH}}_{4}$ emission events, with the MLP model closely matching LI-COR reference data and accurately identifying concentration peaks under diverse atmospheric conditions. Although minor performance degradation was observed under rapid airflow, the system maintained robust operation across multiple environments.

Future work will focus on extending the training dataset to cover broader temperature, humidity, and pressure ranges, enabling improved generalization under varying conditions. Additional developments will explore model structure optimization, adaptive recalibration for long-term drift correction, and integration of lightweight edge inference for on-device model execution(e.g., TensorFlow Lite or TinyML). Furthermore, future iterations will enhance network scalability through low-power wide-area communication (e.g., LoRaWAN or mesh networking) and implement cloud-based analytics for autonomous, distributed CH4 monitoring and source localization [47].

## Figures and Tables

**Table 1:** Comparison of model performance in terms of $R^{2}$ score for validation and test datasets.

Model	Data-Val ($R^{2}$)	${\mathrm{Data-Test}}_{1}$ ($R^{2}$)
Multiple Linear Regression (MLR)	0.912	0.805
Elastic Net Regression (ENR)	0.921	0.919
Support Vector Regression (SVR)	0.935	0.887
Random Forest Regression (RFR)	0.948	0.942
CatBoost Regression (CBR)	0.954	0.930
Multilayer Perceptron (MLP)	0.957	0.948

**Table 2:** Comparison of model performance in terms of RMSE for Balcony Test.

Situation	Elastic Net (RSME)	Multilayer Perceptron (RMSE)
Sunny Day (noon)	1.24 ppm	0.92 ppm
Night	1.44 ppm	1.57 ppm
Raining Day	2.73 ppm	1.17 ppm
Thunderstorm	3.91 ppm	1.55 ppm

## Data Availability

Data are contained within the article.
